# Ibrutinib inhibits antibody dependent cellular cytotoxicity induced by rituximab or obinutuzumab in MCL cell lines, not overcome by addition of lenalidomide

**DOI:** 10.1186/s40164-019-0141-1

**Published:** 2019-08-06

**Authors:** Alexandra Albertsson-Lindblad, Catja Freiburghaus, Mats Jerkeman, Sara Ek

**Affiliations:** 1 Department of Oncology, Skåne University HOSPITAL, Lund University, Lasarettsgatan 23, 221 85 Lund, Sweden; 2 Department of Immunotechnology, Lund University, Lund, Sweden

**Keywords:** Mantle cell lymphoma, Antibody-dependent cell death, Ibrutinib, Lenalidomide, CD20 antibody

## Abstract

**Background:**

The Bruton’s Tyrosine Kinase (BTK)-inhibitor ibrutinib is highly active in mantle cell lymphoma (MCL) but may inhibit response to anti-CD20 antibody as previously shown in CLL models. We investigated how antibody-dependent cellular cytotoxicity (ADCC) induced by type I/II anti-CD20 antibodies was affected by treatment with ibrutinib in MCL. Furthermore, we investigated if lenalidomide, a potential sensitizer to anti-CD20 treatment, could prevent an inhibitory effect of ibrutinib.

**Methods:**

Anti-CD20 (rituximab/obinutuzumab) opsonized MCL cell lines were co-cultured with ibrutinib (± lenalidomide)—exposed effector cells, and analyzed for evaluation of cell death.

**Results:**

Cell death induced by rituximab was reduced with 75% at 0.5 µM ibrutinib and with 52% at 0.1 µM ibrutinib when induced by obinutuzumab, even by addition of lenalidomide. Moreover, obinutuzumab was associated with higher rate of cell death compared to rituximab.

**Conclusion:**

Ibrutinib negatively affects anti-CD20 induced cell death in MCL, not reversed by lenalidomide. Explorations of sequential administration and selective BTK-inhibitors may reveal the optimal combination of novel agents in MCL.

**Electronic supplementary material:**

The online version of this article (10.1186/s40164-019-0141-1) contains supplementary material, which is available to authorized users.

## Background

The addition of anti-CD20 monoclonal antibody (mAb) to chemotherapy has improved disease control and survival rates in mantle cell lymphoma (MCL) and constitutes a backbone in primary treatment [1]. Still, MCL is regarded an incurable disease and exploration of new combinatory regimens is warranted. Ibrutinib, an inhibitor of Bruton's Tyrosine kinase is highly active in MCL and currently combined with anti-CD20 mAb within clinical trials. However, preclinical models in chronic lymphocytic leukemia (CLL) have demonstrated that ibrutinib has a negative impact on the response to anti-CD20 mAb by inhibiting NK cell activation and thereby reducing antibody-dependent cellular cytotoxicity (ADCC) [2, 3].

Consequently, we hypothesized that ibrutinib inhibits immune mediated cell death induced by a type I/II anti-CD20mAb in MCL in vitro (i). Furthermore, we aimed to investigate whether the reduced response to anti-CD20 mAb treatment by ibrutinib could be prevented by pretreatment of effector cells with the immune modulator lenalidomide (ii). Third, we compared the potency of inducing immune mediated cell death between type I and II anti-CD20 mAb bodies (iii).

## Methods and results

To investigate whether ibrutinib affects the immune-mediated response to anti-CD20 mAb in MCL, we performed experiments using the combination in vitro (i). Two well-characterized MCL cell lines, with low sensitivity to ibrutinib (Jeko-1, REC-1) (Additional file 1: Figure S1) were used as target cells, to enable evaluation of the effect of ibrutinib on the cytotoxic activity of peripheral blood mononuclear effector cells (PBMC) and not the direct effect on target cells. PBMC were treated with ibrutinib (0.1/0.5/1/5 µM) or R10 (control) 1 h and incubated with anti-CD20 mAb-coated [rituximab/obinutuzumab (1 µM)] target cells o/n. Target cells were stained with carboxyfluorescein succinimidyl ester (CFSE) at baseline and 7-AAD was used as a marker for the non-viable population (see Additional file 1 for details in Methodology). CFSE and 7-AAD positive (^+^) cells were measured using flow cytometry, and the fraction of non-viable target cells were calculated from the ratio 7-AAD^+^ out of CFSE^+^ populations.

Immune-mediated cell death, hereafter named as cell death, was defined as mean value of (7-AAD/CFSE)-ratio of duplicates compared to mean value of control duplicates without ibrutinib from four individual experiments. Student’s unpaired t test was performed to identify significant differences. A p-value < 0.05 was considered significant.

**Fig. 1:**
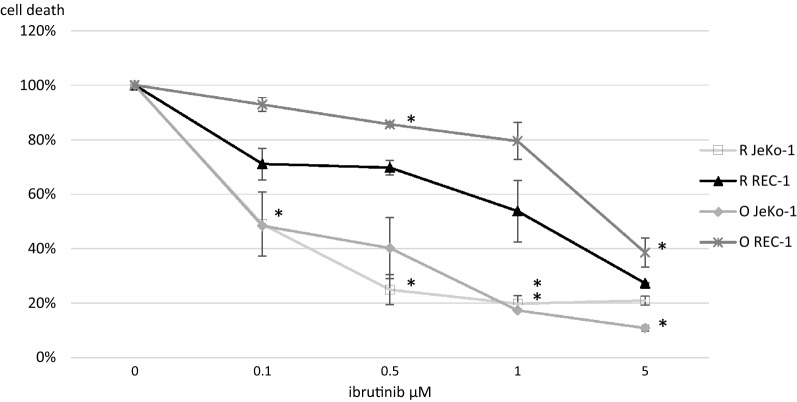
Ibrutinib interferes with immune mediated cell death induced by anti-CD20 antibody in MCL cell lines. Cell death (%) ± standard deviation in MCL cell lines (JeKo -1, REC-1), opsonized with 1 μM anti-CD20 mAb (rituximab, obinutuzumab) and co-cultured with PBMC pretreated with ibrutinib (0-5 µM). Results were compared by unpaired student’s t-test. * = p<0.05. *R* rituximab, *O* obinutuzumab

We found that ibrutinib inhibits ADCC in a concentration dependent manner as shown in Fig. 1 and Table 1. In samples treated with the type I antibody rituximab, a significant lower cell death was observed at ≥ 0.5 µM ibrutinib (0.25 ± 0.06, p = 0.024, JeKo-1) and at 5 µM ibrutinib (0.27 ± 0.00, p = 0.001, REC-1). The maximal difference in cell death was observed at 1 µM ibrutinib (0.20 ± 0.03, p = 0.005, JeKo-1). In samples treated with obinutuzumab, a significant lower cell death was observed at 0.1 µM (0.48 ± 0.30, p = 0.004, JeKo-1) and 0.5 µM (0.86 ± 0.01, p = 0.044, REC-1,) and maximal reduction in cell death was observed at 5 µM ibrutinib (0.11 ± 0.01, p = 0.008, JeKo-1) (0.39 ± 0.05, p = 0.056, REC-1).

**Table 1:** Cell death in MCL cell lines, exposed to anti-CD20 antibody and PBMC pre-treated with ibrutinib

Ibrutinib (µM)	Cell death (± SD)	p-value	Cell death (± SD)	p-value
JeKo-1	Rituximab (1 µM)		Obinutuzumab (1 µM)	
0.1	0.49 ± 0.12	0.145	0.48 ± 0.00	0.003*
0.5	0.25 ± 0.06	0.023*	0.40 ± 0.11	0.118
1	0.20 ± 0.03	0.024*	0.17 ± 0.06	0.005*
5	0.21 ± 0.02	0.012*	0.11 ± 0.01	0.008*
REC-1	Rituximab (1 µM)		Obinutuzumab (1 µM)	
0.1	0.71 ± 0.06	0.126	0.93 ± 0.03	0.222
0.5	0.70 ± 0.03	0.056	0.86 ± 0.01	0.044*
1	0.54 ± 0.11	0.152	0.79 ± 0.07	0.204
5	0.27 ± 0.00	0.001*	0.39 ± 0.05	0.056

* p < 0.05, student’s t-test, compared to control

To investigate whether the immune modulator lenalidomide could revert the repressing effect of ibrutinib on PBMC (ii), the protocol used in (i) was extended by pre-treatment of PBMC with lenalidomide (0/0.01/0.05/0.1/1 µM) 2 h prior to the addition of ibrutinib (1 µM). As in previously described experiments, cell death was lower by pretreatment of PBMC with ibrutinib, but not affected by addition of lenalidomide (Additional file 1: Figure S2 and Table S1). Hence, lenalidomide failed to revert the inhibitory effect of ibrutinib in this experimental set-up. Of note, although there were a trend towards increased cell death at higher concentrations in JeKo-1, we did not observe a significant increased cell death by pretreatment of PBMC with lenalidomide compared to controls (Additional file 1: Figure S3).

**Fig. 2:**
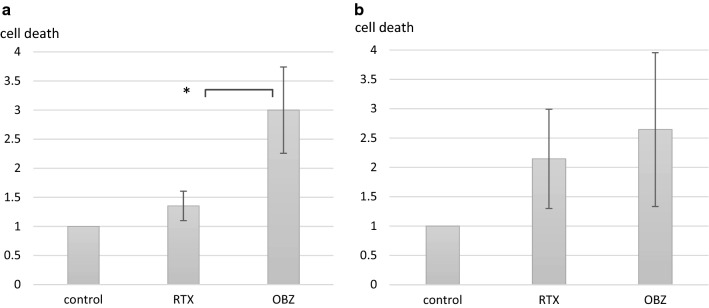
Obinutuzumab induces higher rate of cell death compared to rituximab in MCL cell lines. Cell death of MCL cell lines (JeKo -1, REC-1), treated with CD20-ab [rituximab, obinutuzumab (1 µM)] and co-cultured with PBMC. Data shown are mean values ± standard deviation of cell death from three individual experiments compared to control (no anti-CD20 mAb). **a** JeKo-1, **b** REC-1. *RTX* rituximab, *OBZ* obinutuzumab. Results were compared with student t-test. * = p<0.05

Third (iii), we compared the potency of type I versus type II anti-CD20-mediated ADCC on MCL in vitro, by comparing cell death, measured by 7-AAD^+^/CFSE^+^-ratios of samples with and without anti-CD20 mAb in three individual experiments. We found a significant higher cell death after treatment with obinutuzumab compared to rituximab in one out of two cell lines (Fig. 2 and Table 2).

**Table 2:** Cell death induced by rituximab and obinutuzumab in MCL

	Rituximab		Obinutuzumab		Rituximab vs obinutuzumab
	Cell death (%)	p-value	Cell death (%)	p-value	p-value
JeKo-1	1.31. ± 0.26	0.024*	3.00 ± 1.02	0.012*	0.030*
REC-1	2.15 ± 0.85	0.029*	2.64 ± 1.31	0.038*	0.493

* p < 0.05

In summary, the results of our study demonstrate an inhibitory effect of ibrutinib on immune mediated cell death induced by type I or II anti-CD20 monoclonal antibody treatment in MCL in vitro, not overcome by pre-sensitizing effector cells with the immune modulator lenalidomide. Our findings suggest a more pronounced immune mediated cell death induced by a type II anti-CD20 mAb obinutuzumab than the type I CD20 targeting antibody rituximab.

## Discussion

Ibrutinib is an attractive component in combinatory regimens in MCL, based on its high anti-proliferative activity and favorable toxicity profile. However, little is known about how ibrutinib interferes with the immune-mediated activity induced by anti-CD20 targeted treatment in MCL, which was the focus of this study.

We observed a reduced immune mediated cell death induced by rituximab at physiologically relevant concentrations of ibrutinib (0.1 µM), being within the range of plasma concentration (0.07–0.2 µM) reported in patients receiving 420–840 mg daily ibrutinib [4, 5].

Our results are in line with previous data on in vitro models in CLL [2, 3, 6], which may be explained by affinity of ibrutinib to interleukin-2-inducible T-cell kinase (ITK), expressed in NK cells, but could also be related to reduced CD20-expression [7, 8]. Interestingly, Kohrt et al. demonstrated a superior tumor control by sequential administration of anti-CD20 mAb and ibrutinib compared to concomitant administration of these drugs in ex vivo models in CLL that would be interesting to explore in MCL [2].

We used two cell lines with low and intermediate sensitivity to ibrutinib to minimize the dose-dependent toxic effect on target cells by ibrutinib per se, although a direct cytotoxic effect could not be neglected at 5 μM in one of the cell lines (REC-1), thus possibly overestimating cell death. PBMC was incubated with ibrutinib for 1 h, identical to previous reported works which should be sufficient to reach occupancy of target binding [3]. The concentration of anti-CD20 mAb was based on a pre-study where we observed immune mediated cell death at 1 μM, in line with previous ADCC in vitro assays in NHL [2, 3]. Although being lower than the serum maximal concentration (Cmax) of 3.2 μM in patients after repeated doses of rituximab, 1 μM should thus be adequate to evaluate the hypothesis of the actual work [9].

The use of PBMC instead of purified NK cells, being more alike the in vivo situation, required a high ratio of effector: target cells (100:1) resulting in a low count of target cells in flow cytometry, being a limitation of the study.

In the present study, the inhibitory effect of ibrutinib was not reverted by pre-treatment of PBMC with lenalidomide. Lenalidomide has been reported to potentiate anti-CD20 induced ADCC in vitro but not in in vivo xenograft model [10, 11]. We did not observe a significant higher cell death in control samples with lenalidomide and anti-CD20 mAb compared to anti-CD20 mAb which should be emphasized. A shorter incubation time and no addition of interleukins such as IL-2 and IL-12 may explain a less pronounced activation of effector cells and potential synergistic actions in our set-up. We included concentrations of lenalidomide 0.01–1 μM, being within the range of dose-dependent cytotoxic effect of lenalidomide in previous in vitro experiments and close to the serum concentration of 1.7 μM at oral daily dose of 25 mg [9, 12]. In our experiment, controls included series with lenalidomide-treated PBMC and non-exposed target cells. In these samples, a markedly lower cell death was observed for lenalidomide compared to samples with anti-CD20 mAb (data not reported). Altogether, this clearly demonstrate that treatment with monoclonal antibody is responsible for the major cytotoxic activity in MCL and that immune modulator such as lenalidomide may increase cell death to a limited extent.

Clinical trials have demonstrated that the combination of lenalidomide and rituximab (R2) is active in MCL with a response rate higher than 50% even in patients with relapsed disease [13, 14]. However, pretreatment of lenalidomide to rituximab does not seem to restore sensitivity in rituximab-refractory MCL, according to results from the phase II trial by Chong et al. [15]. Furthermore, ibrutinib-lenalidomide-rituximab was associated with high response rate in relapsed/refractory MCL including patients with poor prognostic risk factors as reported from a Nordic trial [12, 16]. Altogether, the synergistic effects of lenalidomide on anti-CD20 mAb activity in MCL seems to be restricted to anti-CD20-sensitive cases. To further evaluate the role of lenalidomide when combined with rituximab and a BTK-inhibitor such as ibrutinib, it would be valuable to explore how immune mechanisms including NK cell activation status are affected during this combination and if an alternative approach, i.e. by sequential administration, would overcome possible counteracting effects.

Our results suggest that obinutuzumab may have a more pronounced capacity of inducing ADCC in MCL compared to rituximab. These results confirm previous in vitro studies in CLL and other non-Hodgkin lymphoma models [3, 17]. Ongoing clinical trials evaluating the combination of obinutuzumab and ibrutinib are underway and will, together with further preclinical investigations, reveal which is the optimal anti-CD20 targeting agent in MCL [18].

Being a strict in vitro study, our study has several limitations. The results need to be confirmed by other methods for evaluating immune mediated cell death as well as by in vivo experiments to support the hypothesis.

In summary, we show that ibrutinib inhibits the immune mediated response induced by anti-CD20-mAb with markedly lower cell death at concentrations comparable to in vivo serum levels, not overcome by addition of a potential immune sensitizer such as lenalidomide. Although a type II anti-CD20 mAb may have a stronger capacity of inducing immune mediated cell death in MCL, the inhibitory effect of ibrutinib on effector cells seems to be relevant irrespectively if combined with a type I or II anti-CD20 mAb. Besides confirming studies on in vivo models, future studies on sequential administration of ibrutinib and anti-CD20-mAbs in MCL as well as exploration of more selective BTK-inhibitors with less off-target binding may reveal how these optimally could be combined in vivo, with respect to efficacy, potential synergism and toxicity of each compound.

## Conclusion

The findings of our study strongly indicate that the inhibitory effect of ibrutinib on immune-mediated cell death induced by anti-CD20 mAb due to unwanted off-target binding is applicable even in MCL. The inhibitory effect of ibrutinib on effector cells seems to be strong enough to withstand either a more potent activator of effector cells such as a type II anti-CD20 mAb like obinutuzumab or by pretreatment of lenalidomide, a potential sensitizer to anti-CD20mAb. In the light of the high activity of these agents in MCL, sequential administration of the compounds as well as exploring more selective BTK-inhibitors could be of relevance in future design of regimens.

## Additional file

**Additional file 1.** Supplementary tables and figures & Methodology and Material.

## Data Availability

The datasets included in the current study are available from the corresponding author upon reasonable request.

## Abbreviations

7-AAD: actinomycin D
ADCC: antibody-dependent cellular cytotoxicity
BTK: Bruton’s Tyrosine Kinase
CFSE: carboxyfluorescein succinimidyl ester
CLL: chronic lymphocytic leukemia
ITK: interleukin-2-inducible T cell kinase
mAb: monoclonal antibody
MCL: mantle cell lymphoma
NK cell: natural killer cell
PBMC: peripheral blood mononuclear cell
