# Supplementary material for: Ibrutinib inhibits antibody dependent cellular cytotoxicity induced by rituximab or obinutuzumab in MCL cell lines, not overcome by addition of lenalidomide
Source: Exp Hematol Oncol. 2019 Aug 6;8:16. doi: 10.1186/s40164-019-0141-1 (PMC6685275; doi:10.1186/s40164-019-0141-1)
Supplement: Supplementary file 1 — Additional file 1. Supplementary tables and figures & Methodology and Material. [file 40164_2019_141_MOESM1_ESM.docx]

Additional file

Additional material

**Additional tables and figures**

**Additional Figure S1. Direct effect on MCL cell lines (JeKo-1;REC-1).**Mean values of 7-AAD+/CFSE+ cells (±st.dev) for Jeko and REC treated with ibrutinib (0-10uM) o/n followed by calculation of 7-AAD/CFSE+ cells as a marker of cell death from analysis in flow cytometry. *=p<0.05 compared by student´s t test.


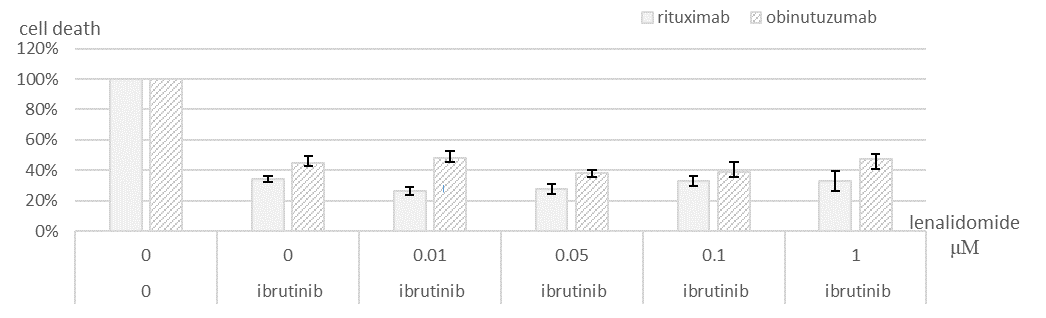


*

*

**Additional Figure S2.** **Lenalidomide does not overcome the inhibitory effect on ADCC induced by anti-CD20-monoclonal antibody.** PBMC pretreated with lenalidomide (0-1 µM) 2 h, followed by addition of ibrutinib 1 µM/1 h and co-cultured with MCL cell line (JeKo-1) exposed for rituximab or obinutuzumab (1 µM). Data shown is mean values of triplicates ± standard deviation compared to control from experiment with one representative donor of PBMC. a) rituximab, b) obinutuzumab. The significantly lower cell death observed in samples treated with ibrutinib, compared to control, was not affected by addition of lenalidomide. Samples were compared by unpaired student t-test. *=p<0.05

|  | cell death (%)±SD | p-value* | cell death (%)±SD | p-value** | cell death (%)±SD | p-value** | cell death (%)±SD | p-value** | cell death (%)±SD | p-value** |
| --- | --- | --- | --- | --- | --- | --- | --- | --- | --- | --- |
| lenaliomid (μM) | 0 |  | 0.01 |  | 0.05 |  | 0.1 |  | 1 |  |
| rituximab | 34.02±2.11 | 0.0005 | 26.30±2.54 | 0.0313 | 27.72±3.08 | 0.0837 | 32.89±3.05 | 0.6924 | 32.80±6.70 | 0.8255 |
| obinutuzumab | 44.59±4.92 | 0.0039 | 47.83±4.65 | 0.5356 | 38.30±1.99 | 0.2046 | 38.60±6.73 | 0.3719 | 47.62±3.19 | 0.5125 |

**Additional Table SI. Lenalidomide does not overcome the inhibitory effect on ADCC induced by anti-CD20-monoclonal antibody.** Mean value of cell death from triplicates ± standard deviation compared to control from experiment with one representative donor of PBMC. *=p-value from comparison with control, **=p-value from comparison with samples with anti-CD20 mAb and ibrutinib.


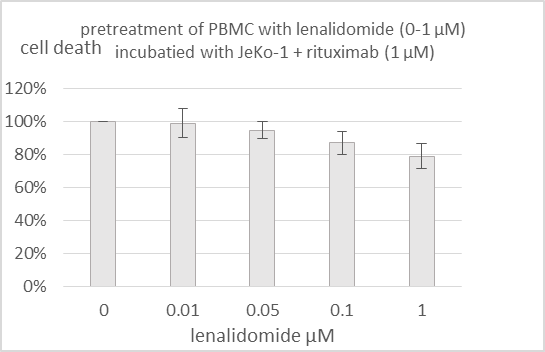

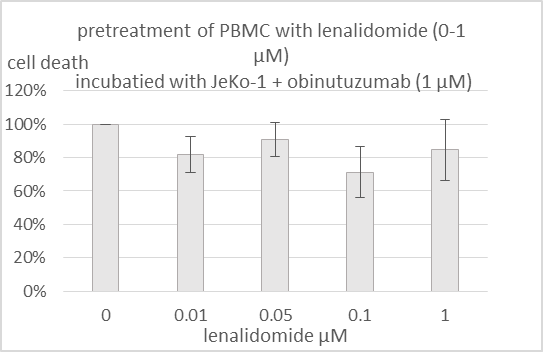


**Additional Figure S3.** **Effect on cell death in rituximab/obinutuzumab-exposed MCL cells (JeKo-1) after pretreatment of PBMC with lenalidomide.** Mean value of cell death from triplicates ± standard deviation compared to control from experiment with one representative donor of PBMC. *=p-value from comparison with control, **=p-value from comparison with samples with anti-CD20 mAb and ibrutinib.

# **Methodology and Material**

## Assessment of immune mediated cell death

Peripheral Blood Mononuclear cells (PBMC) were seeded in 96-w-plate and treated with 0.1/0.5/1/5 µM ibrutinib for 1 h, 37°C, 5% CO2. In parallel, two MCL cell lines, Jeko-1 and REC-1, with low/intermediate sensitivity to ibrutinib per se (as shown in separate experiments, Suppl Figure 1), were used as target cells. Cells were stained with carboxyfluorescein succinimidyl ester (CFSE) (Cell TraceTM Cell Proliferation Kit, Molecular Probes®, ref C34554) for 30 min, RT, washed and incubated with 1 µM anti-CD20-mAb (rituximab/obinutuzumab) for 20 min, 37°C, 5% CO2, and then co-cultured with PBMC o/n 37°C, 5% CO2 with effector:target cell ratio 100:1 . On day 2, cells were washed and stained with 5% 7-Amino-Actinomycin D (7-AAD) (BD Biosciences, ref. 559925), 5 min, RT, to allow identification of non-viable cells, followed by dilution in MACS buffer (PBS/0.5%BSA/2.5%EDTA) to a final well volume of 120 µl prior to flow cytometry.

In (iii), we performed extended experiments on JeKo-1 with pre-treatment of PBMC with lenalidomide 0/0.01/0.05/0.1/1 µM for 2 h, 37°C, 5% CO2, prior to the addition of 1 µM ibrutinib. Subsequent procedure was identical as described in previous section. Each experiment included both cell lines, both rituximab and obinutuzumab and was made with PBMC from four donors to ensure a biological representation of the results. Analyses were made in Microsoft, Excel 2013.

The study was conducted according to protocols approved by local institutional review board in accordance with the Declaration of Helsinki.

## Calculation of immune mediated cell death and statistics

The fraction of 7-AAD-positive out of CFSE-positive cells was calculated (7-AAD+/CFSE+- ratio), representing the ratio of non-viable target cells. Samples with a count of CFSE-positive cells < 230 or of 7-AAD-positive cells < 60 were excluded from further analysis.

The level of immune-mediated cell death, hereafter named as cell death, was defined as mean value of (7-AAD+/CFSE+)-ratio of duplicates with reagents ((i) (ibrutinib);(ii) (ibrutinib and lenalidomide)), compared to mean value of control duplicates without reagents from four individual experiments. When evaluating type I and II anti-CD20 mAb, cell death was defined as the 7-AAD+/CFSE+- ratio of samples with anti-CD20 mAb compared to samples without anti-CD20 mAb from three individual experiments.

Student’s unpaired t test was performed to identify significant differences. A p-value < 0.05 was considered significant.

## Cell cultures

Cells were cultured in R10 (RPM1640 (HyClone Laboratories, Utah, USA) supplemented with 10 % fetal bovine serum (FBS) (Invitrogen, Carlsbad, CA, USA), and 1 % L-glutamate (Invitrogen). Heat-inactivated FBS was used for the ADCC assays. Fresh whole blood was achieved from voluntary healthy donors at the Department of Transfusion Medicine (Skåne University Hospital, Lund, Sweden). Peripheral blood mononuclear cells (PBMC) were achieved from buffy coat by Ficoll-paque (GE Healthcare, Little Chalfont, UK) density gradient centrifugation, cryopreserved in 50% R10/40% FBS/10%DMSO) and stored in -80°C prior to usage within experiments.

## Antibodies and reagents

Ibrutinib (PCID-32765 Selleck Chemicals, Houston, TX, USA), aliquoted in DMSO as 10 mM. Lenalidomide (PCID-216326 Santa Cruz Biotechnology, USA), solved in DMSO to 100 mg/mL, aliquoted as 10 mM. Rituximab (1.3 mg/mL) and obinutuzumab (4 mg/mL) were obtained from Roche (Basel, Switzerland). All reagents were diluted in R10 into desired concentrations.

## Flow cytometry

Flow cytometry was performed using iQue screener (Intellicyt, Albuquerque, NM, USA). The protocol for collection of cells was: sipping time 55s, up-time 5 s with shaking (800rpm) of plate before and after every 6 well during sampling.

Data analyses were performed in Forecyte® Standard Edition 5.2 (Intellicyt). Gating procedure was made after compensation, based on FSC-H and SSC-H, to identify singlets and live cells. Gates for CFSE- and 7-AAD-positive cells were made in channel BL-1 and BL-4 respectively with unstained samples as negative controls. Samples with a count of 7-AAD-positive cells < 60 or of CFSE-positive cells < 230 were excluded from further analysis.
